# The Effects of Computed Tomography with Iterative Reconstruction on Solid Pulmonary Nodule Volume Quantification

**DOI:** 10.1371/journal.pone.0058053

**Published:** 2013-02-27

**Authors:** Martin J. Willemink, Jaap Borstlap, Richard A. P. Takx, Arnold M. R. Schilham, Tim Leiner, Ricardo P. J. Budde, Pim A. de Jong

**Affiliations:** 1 Utrecht University Medical Center, Department of Radiology, Utrecht, The Netherlands; 2 Gelre Hospital, Department of Radiology, Apeldoorn, The Netherlands; The University of Chicago, United States of America

## Abstract

**Background:**

The objectives of this study were to evaluate the influence of iterative reconstruction (IR) on pulmonary nodule volumetry with chest computed tomography (CT).

**Methods:**

Twenty patients (12 women and 8 men, mean age 61.9, range 32–87) underwent evaluation of pulmonary nodules with a 64-slice CT-scanner. Data were reconstructed using filtered back projection (FBP) and IR (Philips Healthcare, iDose^4^-levels 2, 4 and 6) at similar radiation dose. Volumetric nodule measurements were performed with semi-automatic software on thin slice reconstructions. Only solid pulmonary nodules were measured, no additional selection criteria were used for the nature of nodules. For intra-observer and inter-observer variability, measurements were performed once by one observer and twice by another observer. Algorithms were compared using the concordance correlation-coefficient (p_c_) and Friedman-test, and post-hoc analysis with the Wilcoxon-signed ranks-test with Bonferroni-correction (significance-level p<0.017).

**Results:**

Seventy-eight nodules were present including 56 small nodules (volume<200 mm^3^, diameter<8 mm) and 22 large nodules (volume≥200 mm^3^, diameter≥8 mm). No significant differences in measured pulmonary nodule volumes between FBP, iDose^4^-levels 2, 4 and 6 were found in both small nodules and large nodules. FBP and iDose^4^-levels 2, 4 and 6 were correlated with p_c_-values of 0.98 or higher for both small and large nodules. P_c_-values of intra-observer and inter-observer variability were 0.98 or higher.

**Conclusions:**

Measurements of solid pulmonary nodule volume measured with standard-FBP were comparable with IR, regardless of the IR-level and no significant differences between measured volumes of both small and large solid nodules were found.

## Introduction

Since the widespread introduction of chest computed tomography (CT), pulmonary nodules have become a common incidental finding [1]. Despite the fact that the vast majority of pulmonary nodules are benign [2], they are important radiographic predictors for lung cancer and pulmonary metastases. Although some nodules show typical benign characteristics (e.g. calcifications, diameter smaller than 4 mm, peri-fissural location) [3]–[5], nodule size and growth rate remain the most important imaging predictors for malignancy [6]. Therefore, accurate measurements of nodule size at baseline and growth at follow-up CT are important to differentiate between benign and malignant nodules. Pulmonary nodule size can be calculated using diameter measurements or semi-automated nodule volumetry. Semi-automated software is the preferred method since its repeatability and ability to detect growth is superior to manual two-dimensional diameter measurements [7].

Currently, CT images are reconstructed using standard filtered back projection (FBP). Other reconstruction algorithms for CT imaging have been developed, such as iterative reconstruction (IR). IR algorithms reduce noise and artifacts which potentially can be used to lower CT radiation dose [8]–[11], or to improve image quality at constant dose [12]; [13]. With most IR algorithms the level of noise reduction can be selected, higher levels result in stronger noise reductions [14]. Several in-vivo studies focused on dose reduction and volumetric analysis of pulmonary nodules [15]; [16]. For solid pulmonary nodules no dose dependency was found and volumetry performed equally well on normal-dose and low-dose CT reconstructed with FBP [15]; [16]. However the overall image quality of FBP reconstructed chest CTs decreases with low-dose acquisitions [17], suggesting that IR may be a good option to improve overall image quality of low-dose chest CT scans which is important for evaluation of additional pathological findings. Nevertheless, the in-vivo effects of IR on pulmonary nodule volume measurements have not been systematically investigated and it may be that IR introduces systematic differences in lung nodule volumetry compared to FBP. Therefore, the objective of this study was to compare solid pulmonary nodule volumes measured with semi-automatic software at standard FBP and multiple IR levels.

## Materials and Methods

### Subjects

Twenty patients (12 women, mean age 61.9±12.0 (standard deviation) years) who underwent a CT of the chest between August 2011 and December 2012 were selected by one observer during clinical duty. These patients were retrospectively included in the study if pulmonary nodules were present. The study population is not a truly consecutive sample of all patients with nodules during the study period. The Utrecht Medical Research Ethics Committee approved this study and agreed to a waiver. Informed consent was waived as the study retrospectivelly used CT scans obtained in routine care in anonymous fashion and no additional CT scans were obtained for the purpose of this study. CT scans were reconstructed using FBP and different IR levels. Scan indications were suspected or known lung carcinoma (N = 11), lung metastases (N = 8) and neuroendocrine lung tumor (N = 1).

### CT protocol

Image acquisition was performed on a 64-slice scanner (Brilliance 64, Philips Healthcare, Best, The Netherlands) with moderate edge enhancing reconstruction filter C. Patients underwent a contrast-enhanced chest CT. Image acquisition depended on indication and body weight; tube voltages were either 100 kVp (N = 10) or 120 kVp (N = 10), with a median tube current-time product of 100 mAs (quartile range 77–115 mAs). Reconstructed slice thickness was 0.9 mm with 0.7 mm increment. Volume CT dose index (CTDI_vol_) and dose length-product (DLP) were recorded for each CT exam. An estimate of the effective dose was calculated by multiplying the DLP with the effective dose estimate of 0.0145 mSv/(mGy×cm) for the chest [18].

### Image reconstruction

Reconstruction of raw data was performed using standard FBP and three levels of a commercially available IR algorithm (iDose^4^, Philips Healthcare, Best, the Netherlands) [13]; [19]–[21]. IR algorithms are developed to allow reduction of CT radiation dose by reducing noise and artefacts. The iterative process comprises the optimization of measured raw data or image data based on the noise reducing IR model, and evaluation of the optimized data by comparison with the measured raw data or image data. With every iteration, the image is updated into an image with less noise, resulting in an optimized final image. Currently available IR techniques range from basic algorithms which only reduce noise of the image data to advanced algorithms that fully iterate with forward and backward reconstruction steps [14]; [22]. The version of iDose used in the current study, is the fourth generation of Philips' IR algorithm and reduces noise of both the raw data and the image data. First a Poisson statistics based maximum likelihood denoising algorithm is used to identify and correct the noisiest raw CT data while preserving edges [21]. Subsequently, images are reconstructed from the denoised raw data. Uncorrelated noise in the images is decreased by iterative filtering. Noise reduction level can be adjusted by choosing one of seven levels. Higher levels lead to more noise reduction. We used iDose^4^ levels 2, 4 and 6 in the present study. According to the manufacturer these levels result in 16, 29 and 45% noise reduction, respectively [23].

### Volumetry protocol

Volumetric measurements of pulmonary nodules were performed using commercially available semi-automatic software (IntelliSpace version 4.0.0.40259, Philips Healthcare, Best, the Netherlands) on a dedicated CT workstation. Only solid nodules were measured, no additional selection criteria were used for the nature or location of nodules. Criteria for solid nodules were in accordance with the Fleischner Society and included well or poorly defined rounded or irregular homogenous opacities with a diameter smaller than 3 cm (smaller than approximately 14,000 mm^3^) [24]. All measurements were done using the thinnest reconstructed slices (0.9 mm). The software automatically delineated the nodule and quantified its volume after placing the cursor within the nodule and clicking with the mouse. No manual adjustments were made, because automatic measurements were visually judged acceptable. For assessment of inter-observer and intra-observer variability, all measurements were performed once by two observers (MW and RT), and twice by one observer (MW) with a time interval of one week.

### Data analysis

Volumetric measurements of pulmonary nodules with standard FBP and three IR levels were compared within each subject. Lin's concordance correlation coefficient (p_c_) was used to assess the agreement of measurements, because this method takes into account the correlation as well as the distance to the line of identity [25]. Poor agreement is defined as p_c_ <0.90, whereas higher p_c_-values represent moderate (0.90≤ p_c_ <0.95), good (0.95≤ p_c_ ≤0.99), or excellent (p_c_ >0.99) agreement [26]. Statistical differences of variables within subjects were compared using the Friedman test and post-hoc tests were performed with the Wilcoxon signed ranks test. The statistical significance level of the Friedman test was set at a p-value below 0.05 and for post-hoc tests at a p-value below below 0.017 based on the Bonferonni correction for three comparisons (FBP with level 2, level 4 and level 6, respectively). Measured volume differences of 25% or more were defined to be clinically relevant, based on the findings of De Hoop and colleagues [1]. Values are given as medians with quartile ranges, unless otherwise stated. Statistical analyses were performed by using SPSS version 20.0 (SPSS Inc, Chicago, Illinois, USA) and MedCalc statistical package version 12.2.0.0 (MedCalc Software, Mariakerke, Belgium).

## Results

The mean age of the twenty included subjects (12 female, 60%) was 61.9 (range 32–87) years. The median CTDI_vol_ was 4.3 mGy (3.5–7.1 mGy), the median DLP was 207.8 mGy*cm (156.7–313.1 mGy*cm), and the median effective dose was 3.1 mSv (2.3–4.5 mSv). Within these twenty subjects, 78 nodules were found including 56 small nodules (volume <200 mm^3^, diameter <8 mm) and 22 large nodules (volume ≥200 mm^3^, diameter ≥8 mm). Numbers of nodules per patient ranged from 1 to 15 and therefore nodules are not fully independent. The measured pulmonary nodule volumes are listed in Table 1.

**Table 1:** Results of pulmonary nodule measurements.

	FBP	iDose^4^ L2	p-value	iDose^4^ L4	p-value	iDose^4^ L6	p-value*
**Small nodules** (mm^3^)	77.5 (40.8–127.0)	74.5 (45.8–127.4)	NS	72.8 (43.1–119.0)	NS	73.8 (39.4–120.8)	NS
**Large nodules** (mm^3^)	520.8 (346.2–881.4)	525.0 (363.8–912.7)	NS	526.5 (347.1–870.8)	NS	531.3 (318.0–864.3)	NS

CT measured pulmonary nodule volumes using filtered back projection (FBP) and iterative reconstruction (iDose^4^ level 2, 4 and 6). Values are presented as medians with interquartile range.

*p-value* Based on Wilcoxon signed ranks test; *FBP* Filtered back projection; *L2* Level 2; *L4* Level 4; *L6* Level 6; *NS* Not significant.

### Small nodules

**Figure 1:**
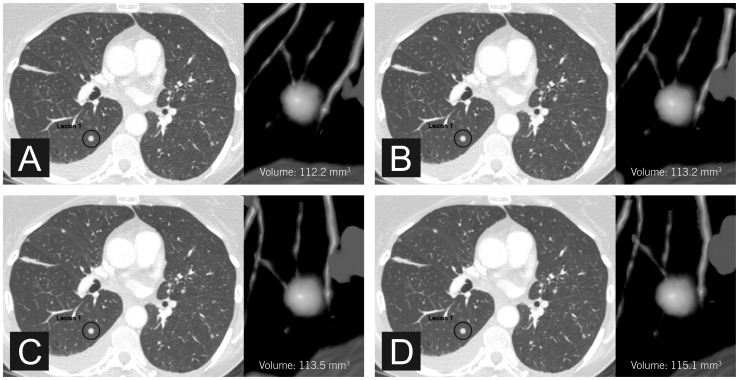
Axial CT images of a small pulmonary nodule. Measured volumes were 112.2 mm^3^ (A, FBP), 113.2 mm^3^ (B, iDose^4^ level 2), 113.5 mm^3^ (C, iDose^4^ level 4), and 115.1 mm^3^ (D, iDose^4^ level 6).

**Figure 2:**
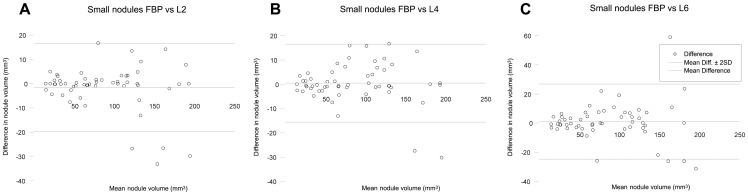
Agreement of small pulmonary nodule measurements. Bland-Altman plots of CT measured nodule volume at filtered back projection (FBP) and iterative reconstruction (iDose^4^ level 2 (A), 4 (B) and 6 (C)) for small nodules. The horizontal axis shows the mean value of the measured nodule volume with FBP and iterative reconstruction. The vertical axis shows the difference between the measured nodule volume with FBP and iterative reconstruction. *FBP* Filtered back projection; *L2* Level 2; *L4* Level 4; *L6* Level 6.

In small nodules (volume <200 mm^3^, diameter <8 mm), the median pulmonary nodule volume at FBP, iDose^4^ levels 2, 4 and 6 was 77.5, 74.5, 72.8 and 73.8 mm^3^, respectively. The Friedman test showed that differences were not significant (p = 0.910). Two small nodules were measured with differences ≥25% compared with FBP (−31% (nodule volume 193.0 mm^3^, iDose^4^ level 6) and +44% (nodule volume 59.8 mm^3^, iDose^4^ level 6)). All IR levels showed substantial agreement with FBP measurements with a concordance correlation coefficient (p_c_) of 0.98 for iDose^4^ level 2, 0.99 for iDose^4^ level 4, and 0.97 for iDose^4^ level 6. Figure 1 shows an example of a small pulmonary nodule. Results of the measured volumes for small nodules are demonstrated in Figure 2.

### Large nodules

**Figure 3:**
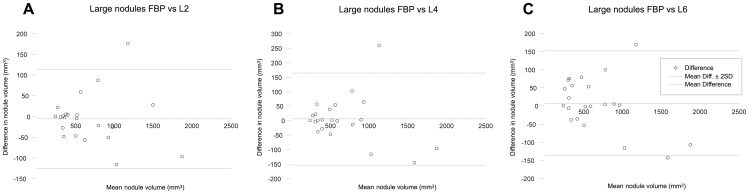
Agreement of large pulmonary nodule measurements. Bland-Altman plots of CT measured nodule volume at filtered back projection (FBP) and iterative reconstruction (iDose^4^ level 2 (A), 4 (B) and 6 (C)) for large nodules. The horizontal axis shows the mean value of the measured nodule volume with FBP and iterative reconstruction. The vertical axis shows the difference between the measured nodule volume with FBP and iterative reconstruction. *FBP* Filtered back projection; *L2* Level 2; *L4* Level 4; *L6* Level 6.

In large nodules (volume ≥200 mm^3^, diameter ≥8 mm), the median pulmonary nodule volume measured with iDose^4^ levels 2, 4 and 6 was 525.0, 526.5 and 531.3 mm^3^, respectively, which was slightly larger compared to FBP (520.8 mm^3^). Again, no significant differences were found between measured nodule volumes at FBP, iDose^4^ level 2, 4 and 6 (p = 0.684). No large nodules were measured with differences ≥25% compared with FBP. Furthermore, all IR levels showed substantial agreement with FBP measurements (p_c_ was 0.99 at iDose^4^ levels 2 and 6, and 0.98 at iDose^4^ level 4). Results of the measured volumes for large nodules are demonstrated in Figure 3.

### Intra-observer and inter-observer variability

The results of the intra-observer and inter-observer variability assessment are listed in Table 2. There were no significant differences between nodule volume measurements of the two observers (all p>0.05) for both small and large nodules. Intra-observer and inter-observer variability were good or excellent for small nodules as well as for large nodules (all p_c_≥0.98).

**Table 2:** Intra-observer and inter-observer variability.

		Small nodules		Large nodules	
		p-value	p_c_-value	p-value	p_c_-value
**FBP**	Observer 1–1 vs observer 1–2	0.068	0.98	0.317	0.99
	Observer 1–1 vs observer 2	0.075	0.98	0.317	0.99
	Observer 1–2 vs observer 2	0.854	1.00	0.317	1.00
**iDose^4^** **L2**	Observer 1–1 vs observer 1–2	0.655	1.00	0.655	1.00
	Observer 1–1 vs observer 2	0.465	1.00	0.317	1.00
	Observer 1–2 vs observer 2	0.593	1.00	0.285	1.00
**iDose^4^** **L4**	Observer 1–1 vs observer 1–2	0.080	0.99	1.000	1.00
	Observer 1–1 vs observer 2	0.109	1.00	0.180	1.00
	Observer 1–2 vs observer 2	0.285	1.00	0.180	1.00
**iDose^4^** **L6**	Observer 1–1 vs observer 1–2	0.500	0.99	0.317	1.00
	Observer 1–1 vs observer 2	0.463	0.99	0.317	1.00
	Observer 1–2 vs observer 2	0.893	1.00	1.000	1.00

P-values and concordance correlation coefficients (p_c_-values) of measured pulmonary nodule volumes using filtered back projection (FBP) and iterative reconstruction (iDose^4^ level 2, 4 and 6) by two observers.

*p-value* Based on Wilcoxon signed ranks test; *p_c_-value* Concordance correlation coefficient; *FBP* Filtered back projection; *L2* Level 2; *L4* Level 4; *L6* Level 6; *Observer 1–1* First measurement by first observer; *Observer 1–2* Second measurement by first observer; *Observer 2* Measurement by second observer.

## Discussion

Iterative CT reconstruction is an interesting method for noise reduction and improving image quality that is now provided on the CT scanners of all major vendors. Previous studies have shown that nodule volumetry is possible on low-dose chest CT and that IR allows for radiation dose reduction of chest CT while image quality is maintained at low-dose CT [8]; [27]; [28]. Thus, it is to be expected that IR will soon be widely implemented for clinical use, but it is unknown whether nodule volumetry is affected by IR. We found that pulmonary nodule volumes measured with standard FBP were comparable with IR, regardless of the IR level and no significant differences between measured volumes of both small solid nodules and large solid nodules were found.

To our knowledge, no in-vivo study has been performed to analyze the effects of IR on pulmonary nodule volume measurements. One study researched the effect of IR on the performance of a pulmonary nodule computer-aided detection system in terms of sensitivity and specificity [29], however the effect on nodule volumetry was not analyzed. Another study evaluated the effect of IR in a phantom on in-vitro lung nodule volumetry, resulting in similar results as our current findings [30]. The finding of the current study that IR does not affect solid pulmonary nodule volume measurements is relevant, since it demonstrates that it is safe to convert FBP protocols to IR for solid pulmonary nodule volumetry. This also accounts for patients who are already in a follow-up scheme. Since reducing the radiation dose affects the overall image quality of FBP reconstructed chest CTs, IR may be applied to reduce the radiation dose without compromising on overall image quality. This is important, because chest CTs are not only used for lung nodule volumetry but also for evaluation of other pathological findings. Our study suggests that IR has no clinically relevant effect on pulmonary nodule volume measurements. This is probably because IR algorithms do not only reduce noise but also preserve edges which prevents tissue borders from becoming blurry. Moreover, contrasts between air and pulmonary tissues are intrinsically large. These volume measurement effects might be different in other parts of the body that have less contrast on CT.

In our study we had some outliers. De Hoop et al. [1] found 16.4% to 22.3% variability in pulmonary nodule volumetry between different scans. A change in measured nodule volume of 25% is exceeding the normal variability of subsequent scans and can be regarded as nodule growth. As IR provides similar results compared to FBP it is expected that the 25% rule is maintained when using IR although we did not study interscan variation. Two small nodules were measured with clinically relevant differences of ≥25% between FBP and IR. Re-measuring these nodules resulted in the same values. The large differences in these small nodules can possibly be explained because minor absolute differences result in large relative differences.

The intra-observer and inter-observer variability of our study was good to excellent. This is in accordance with previous studies who have shown that nodule volume measurements with semi-automatic software have good reproducibility [31]; [32]. Note that the only manual observer interaction with the software was selection of the seed point.

The present study has limitations. CT scanners, IR technique and semi-automatic software package were used from a single vendor and we cannot comment on other vendors or software packages. Therefore, future research with hardware from other vendors is recommended. Furthermore, volumetry was performed on routine chest CTs and the effect of lowering radiation dose on nodule volumetry was not analyzed. Nevertheless, prior work has demonstrated that pulmonary nodule volumetry was not affected by dose [15]; [16]. But future research on the effects of even lower radiation doses with IR would be of great interest.

In conclusion, this study found that CT volumetry of solid pulmonary nodules did not result in clinically relevant differences with iterative reconstruction.
